# Neural pathways that control the glucose counterregulatory response

**DOI:** 10.3389/fnins.2014.00038

**Published:** 2014-02-26

**Authors:** Anthony J. M. Verberne, Azadeh Sabetghadam, Willian S. Korim

**Affiliations:** Clinical Pharmacology and Therapeutics Unit, Department of Medicine, Austin Health Heidelberg, The University of MelbourneMelbourne, VIC, Australia

**Keywords:** glucose sensing, glucagon, adrenaline, rostral ventrolateral medulla, perifornical hypothalamus, ventromedial hypothalamus, counterregulation, hypoglycemia

## Abstract

Glucose is an essential metabolic substrate for all bodily tissues. The brain depends particularly on a constant supply of glucose to satisfy its energy demands. Fortunately, a complex physiological system has evolved to keep blood glucose at a constant level. The consequences of poor glucose homeostasis are well-known: hyperglycemia associated with uncontrolled diabetes can lead to cardiovascular disease, neuropathy and nephropathy, while hypoglycemia can lead to convulsions, loss of consciousness, coma, and even death. The glucose counterregulatory response involves detection of declining plasma glucose levels and secretion of several hormones including glucagon, adrenaline, cortisol, and growth hormone (GH) to orchestrate the recovery from hypoglycemia. Low blood glucose leads to a low brain glucose level that is detected by glucose-sensing neurons located in several brain regions such as the ventromedial hypothalamus, the perifornical region of the lateral hypothalamus, the arcuate nucleus (ARC), and in several hindbrain regions. This review will describe the importance of the glucose counterregulatory system and what is known of the neurocircuitry that underpins it.

## Introduction

Glucose is a major source of energy for all cells in mammals. In particular, the nervous system requires a continuous supply of glucose to support its energy requirements and maintain metabolic homeostasis. A large proportion of energy provided by glucose is used only to support the neuronal resting membrane potential. In addition, marked regional differences in glucose utilization may be associated with changes in cognitive function even at steady state. As such, multifaceted physiological mechanisms were selected for during the evolution of mammalian species to adjust and maintain blood glucose within a narrow range. By contrast, in Type 1 diabetes, pathological increases in blood glucose, known as hyperglycemia, may lead to adverse, chronic consequences including cardiovascular disease, neuropathy, retinopathy and nephropathy. In Type 1 diabetes, hyperglycemia is treated with insulin to restore normoglycemia. However, diabetic patients may also experience hypoglycemia, as a result of inappropriate doses of insulin. Similarly, ~30% of patients with advanced Type 2 diabetes treated with hypoglycemic agents can experience hypoglycemia. If severe, hypoglycemia can result in convulsions, loss of consciousness, coma and even death. In order to restore normoglycemia, the body activates a series of defense mechanisms that act in conjunction and are referred to as the “glucose counterregulatory response.” The autonomic and neuroendocrine responses associated with the glucose counterregulatory response are usually accompanied by other behaviors such as arousal and feeding.

Although the mechanisms that underpin glucose homeostasis reside partly in the periphery, it is apparent that the central nervous system plays an important role in glucose counterregulation. For instance, adrenaline release in response to hypoglycemia or glucoprivation (local glucose deprivation) is essentially mediated by the sympathetic nervous system. Glucose sensors are distributed throughout several bodily regions and are capable of detecting decreases in glucose levels in the plasma and in the brain extracellular milieu. Activation of some of these sensors results in glucose counterregulation by adjusting the secretion of several hormones. In response to declining plasma glucose there is a decrease in insulin secretion and increases in glucagon, adrenaline, cortisol, and GH secretion. Decreases in glucose levels are detected by glucose-sensing neurons that are found in several brain regions including the ventromedial hypothalamus, the perifornical region of the lateral hypothalamus (PeH), the arcuate nucleus (ARC), as well as in several hindbrain regions and in the periphery e.g., pancreas, carotid body, liver, and gastrointestinal tract. This review addresses the central and peripheral neural pathways involved in blood glucose homeostasis.

## Glucose sensing

### Glucose sensing in the periphery

**Figure 1:**
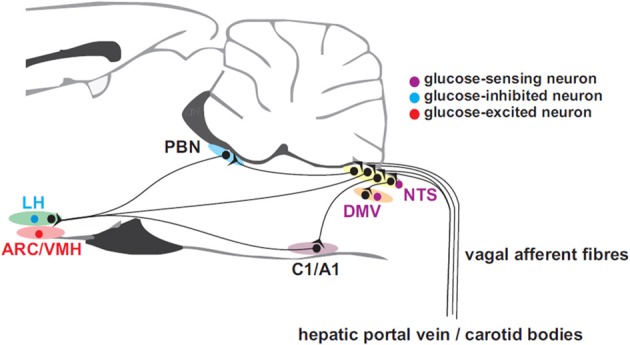
**Afferent inputs from the periphery to brain neurons involved in glucose homeostasis**. Glucose-sensing vagal and sympathetic afferents arise from the liver and gastrointestinal tract and convey information to the nucleus of the solitary tract (NTS). Unlike their sympathetic sensory counter-parts arising near the portal vein, vagal glucose sensors probably do not contribute the counter-regulatory response. Carotid body glucose sensors may also contribute to the counter-regulatory response. Information is relayed from NTS to the dorsal motor nucleus of the vagus (DMV) which provides parasympathetic drive to the pancreatic islets and via the parabrachial nucleus (PBN) top supramedullary structures. C1 adrenergic neurons with projections ascending to hypothalamic sub-regions such as the arcuate (ARC), lateral (LH), and ventromedial hypothalamic (VMH) nuclei, are involved in the feeding response to insulin-induced hypoglycemia.

Apart from glucose-sensing by pancreatic β-cells, which will not be dealt with in this review, peripheral glucose sensing has been demonstrated at several sites including the liver, via the hepatic portal vein, vagal (Adachi, 1981) and sympathetic afferents, intestinal vagal glucose sensors, and possibly the carotid body (Figure 1). Hepatic glucose sensors appear to be necessary for expression of the sympathoadrenal response to hypoglycemia (Donovan et al., 1991) and are located close to or in the portal vein. Portal vein denervation blunts the adrenal catecholamine response to slowly-developing hypoglycemia (Hevener et al., 2000). These portal vein sensory afferents contain calcitonic gene-related peptide since they are capsaicin sensitive, but probably do not travel in the vagus (Fujita et al., 2007). The carotid body may also sense glucose (Pardal and Lopez-Barneo, 2002; Conde et al., 2007; Garcia-Fernandez et al., 2007) and contribute to the counterregulatory modulation of glucagon secretion (Koyama et al., 2000). However, its chemosensitivity to CO_2_ and O_2_ hampers the interpretation of glucose-sensing afferent signals. In man, hyperoxia attenuates the counterregulatory hormonal responses to insulin-induced hypoglycemia (Wehrwein et al., 2010). Nevertheless, the glucose-sensing locus seems to shift from the portal-mesenteric vein to a different site (e.g., central nervous system) during fast developing hypoglycemia (Saberi et al., 2008).

### Glucose sensing in the brain

In the central nervous system glucose levels are necessarily maintained at ~0.4–2.5 mM, in which glucose-sensing involves the interplay of neurons and astrocytes (Marty et al., 2005). Their machinery involves the activity of glucokinase, adenosine triphosphate-sensitive K^+^ (K_ATP_) channels, AMP-activated protein kinase (AMPK), odium-glucose co-transporters, and glucose transporter type 2 (Glut2). The membrane potential of glucosensing neurons changes according to their intracellular metabolism (Oomura et al., 1974; Rowe et al., 1996) and to potentials produced by the interaction of glucose with glucose transporters (O'Malley et al., 2006; Williams et al., 2008). Signaling in glucose-sensing neurons and astrocytes involves glucose uptake by Glut2. Following glycolysis in astrocytes, lactate is produced and released into the extracellular space. Extracellular glucose and lactate from astrocytes are internalized by neurons. Lactate is internalized via monocarboxylate transporter 2 whereas glucose is phosphorylated by glucokinase (Levin et al., 2004) and converted to pyruvate (Lam et al., 2005; Marty et al., 2007).

In glucose excited (GE) neurons (Oomura et al., 1964, 1974), oxidative phosphorylation of glucose and the internalization of lactate by monocarboxylate transporters increases the intracellular ATP/ADP ratio resulting in closure of KATP channels (Lee et al., 1999; Miki et al., 2001). Subsequent membrane depolarization leads to action potential generation resulting in activation of voltage-gated calcium channels and neurotransmitter release (Amoroso et al., 1990; Moriyama et al., 2004). Glucose inhibited (GI) cells (Oomura et al., 1964, 1974) have a glucose-sensing mechanism that involves glucokinase in part. It has been speculated, however, that a rise in the intracellular ATP/ADP ratio results in augmented activity of the Na^+^/K^+^ ATPase pump (Oomura et al., 1974; Song and Routh, 2005). Alternatively, a reduction in extracellular glucose increases intracellular AMP raising the activity of AMPK (Murphy et al., 2009). This mechanism is potentiated by augmented concentrations of guanylate cyclase driven by nitric oxide, which production is stimulated by AMPK. The increase in concentration of AMPK activates the cystic fibrosis transmembrane conductance regulator, increasing chloride conductance and hyperpolarizing the cell (Murphy et al., 2009).

Nonetheless, the presence of the aforementioned transporters, channels, and kinases does not define a glucose sensing neuron. For instance, in the ventromedial hypothalamic nucleus (VMH) ~65% of GE and 45% of GI neurons have their responses gated by glucokinase (Kang et al., 2004). In addition, KATP channels are ubiquitous and contribute to diverse physiological functions. Finally, these mechanisms also fail to explain why neurons in the VMH do not express Fos in response to systemic glucoprivation or hypoglycemia (Briski and Sylvester, 2001; Cai et al., 2001). Hence, electrophysiological characterization is the most effective method for identification of glucosensing neurons.

The role of the hindbrain in glucose sensing and control of the counterregulatory response has been reviewed recently by Ritter et al. (2011). It was proposed that the hindbrain contains all of the elements necessary for orchestration of the counterregulatory response. Glucose counterregulatory responses to neuroglucoprivation remained following decerebration, or obstruction of the cerebral aqueduct in rat (DiRocco and Grill, 1979; Ritter et al., 1981). The evidence for this notion is convincing and it is possible that hypothalamic and hindbrain systems operate cooperatively as redundant or “fail-safe” mechanisms. Glucose-sensing neurons have been identified in the dorsal motor nucleus of the vagus (DMV) and the solitary tract nucleus (NTS) (Adachi et al., 1995). However, these sites do not clearly overlap with sites identified using localized glucoprivation (Andrew et al., 2007). Perhaps this is because the relatively large injection volumes that were used in these studies do not allow fine discrimination of the regions that are sensitive to localized neuroglucoprivation. Early studies that support an important role for the hindbrain (DiRocco and Grill, 1979; Ritter et al., 1981) did not unequivocally identify the participation of the sympathoadrenal system. Nevertheless, immunotoxic destruction of the rostral C1 medullospinal neurons in the RVLM eliminates the hyperglycemia, adrenaline secretion and adrenal medullary Fos expression in response to the glucoprivic agent 2-deoxy-D-glucose (2DG) (Ritter et al., 2001; Madden et al., 2006). This is in agreement with our report that the rostral ventrolateral medulla (RVLM) contains medullospinal neurons that are activated by 2DG and that stimulation of these neurons results in hyperglycemia that is markedly reduced by prior adrenalectomy (Verberne and Sartor, 2010). In the studies that used the immunotoxin it is somewhat surprising that a rise in glucagon secretion did not compensate for the loss of adrenaline secretion in response to 2-DG (Karlsson and Ahren, 1991).

In the forebrain, glucose-sensing occurs primarily in the hypothalamus. A strong case has been made for the importance of the VMH in orchestration of the counterregulatory response to hypoglycemia (Borg et al., 1994, 1995, 1997, 1999; Tong et al., 2007). Glucose-sensing neurons have been identified in the VMH, PeH, and the ARC (Oomura et al., 1974; Burdakov et al., 2005a,b; Routh, 2010). It is likely that these different groups of glucose-sensing neurons subserve different physiological roles that may include the counterregulatory response, energy balance and sensations of hunger.

## Neural circuitry involved in the glucose counterregulatory response

### Glucose control by hypothalamic neurons

The involvement of hypothalamic neurons in blood glucose control has been determined by neuroanatomy, neurochemistry, electrophysiology, and neuropharmacology. Hypoglycemia (Moriguchi et al., 1999; Cai et al., 2001) or systemic glucoprivation (Briski and Sylvester, 2001) excites neurons in the ARC, the paraventricular nucleus (PVN), dorsomedial hypothalamic nucleus (DMH), VMH, and lateral hypothalamus (LH; including the perifornical area), as determined by Fos expression in these neurons. Additionally, some of those neurons were characterized according to their electrophysiological properties in response to changes in glucose levels and glucoprivation (Oomura et al., 1964, 1969, 1974; Burdakov et al., 2005a, 2006; Gonzalez et al., 2008). The majority of the GE neurons are positioned laterally in the hypothalamus, whereas GI neurons are located ventromedially.

Studies using neurotropic viruses have shown that in the perifornical hypothalamus only orexin- and MCH-containing neurons project to adrenal sympathetic premotor neurons in the RVLM (Kerman et al., 2007). In addition, insulin-induced hypoglycemia or neuroglucoprivation induces Fos expression in orexin neurons of the PeH suggesting a possible role in glucose sensing (Moriguchi et al., 1999; Briski and Sylvester, 2001; Cai et al., 2001; Paranjape et al., 2006; Tkacs et al., 2007). On the other hand, an *in vitro* study has shown that GI orexin neurons respond in an identical fashion to both glucose and 2DG through a K^+^ channel-mediated mechanism. In addition, these studies showed that this glucose-sensing mechanism is direct and operates independently of glucose metabolism (Gonzalez et al., 2008). This suggests that the orexin neurons are not the principal glucose-sensors involved in the counterregulatory response. This discrepancy may be explained if the site of action of 2DG may not be directly at the PeH orexin neurons but at some other synaptically connected location. In addition, the complexity of hypothalamic interconnections limit the precision with which we can identify glucose-sensing neurons that modulate the counterregulatory response.

### Hypothalamic descending pathways

**Figure 2:**
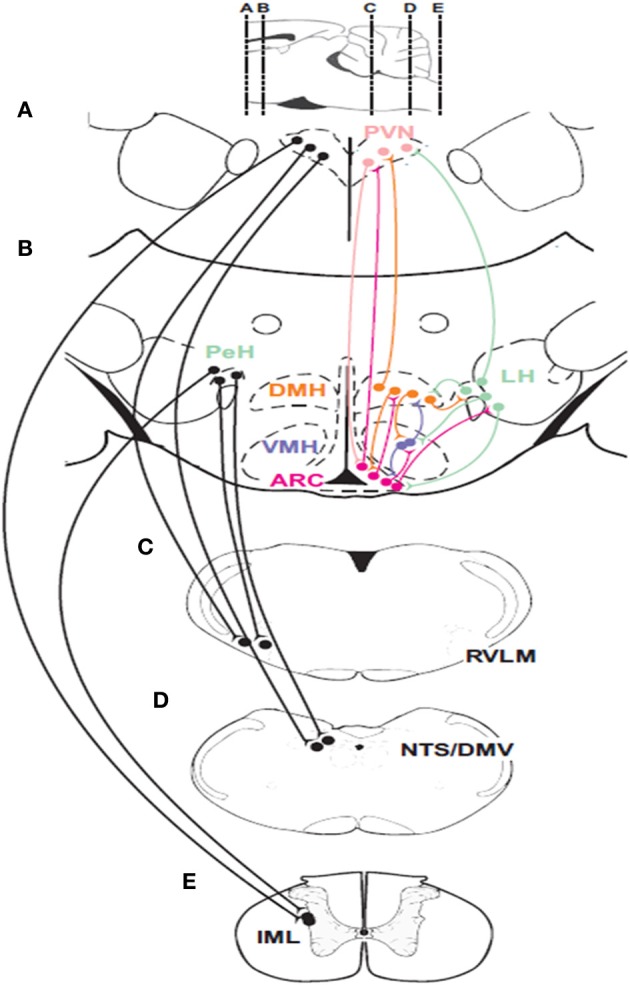
**Descending connections and intrahypothalamic pathways involved in glucose homeostasis**. Neurons in the paraventricular nucleus of the hypothalamus (PVN) and the perifornical region of the hypothalamus (PeH) have connections with important premotor sympathetic and parasympathetic neuronal groups located in the rostral ventrolateral medulla (RVLM) and the dorsal motor nucleus of the vagus (DMV) as well as to the major sensory relay structure the nucleus of the solitary tract (NTS) and sympathetic preganglionic neurons (SPNs) located in the intermediolateral cell column (IML) of the spinal cord. Glucose-sensing neurons are found in the ARC, the ventromedial hypothalamic nucleus (VMH) and the perifornical region (PeH) of the lateral hypothalamic (LH) area. Parasympathetic efferents to the pancreatic islets can activate insulin and glucagon secretion while C1 neurons in the RVLM provide drive to adrenal SPNs. Parasagittal section at the top of the figure indicates rostrocaudal locations of coronal sections **(A–E)**.

Hypothalamic responses to hypoglycemia occur via connections with sympathetic and parasympathetic efferent neurons in the brainstem and spinal cord (Figure 2). Anterograde and retrograde transport studies show that neurons in the PVN and LH project directly to sympathetic preganglionic motor neurons (SPN) in the spinal cord (Saper et al., 1976; Luiten et al., 1985), and catecholaminergic sympathetic premotor neurons (C1) (Ter Horst et al., 1984; Luiten et al., 1985; Allen and Cechetto, 1992; Shafton et al., 1998) in the RVLM. Furthermore, orexinergic and MCH neurons in the LH project to both sympathetic groups (Bittencourt et al., 1992; Peyron et al., 1998; Kerman et al., 2007). However, the evidence for differential sympathetic control of adrenaline and glucagon release is scarce. Although neurotropic viral transport studies (Strack et al., 1989a,b; Kerman et al., 2007) confirm that these pathways are involved in the control of the chromaffin cells, they coincide with the sympathetic pathways that control the pancreas (Jansen et al., 1997). Additionally, the synergism between the PVN and LH extends outside their communication through neural pathways. For example, an increase in circulating adrenaline stimulates corticotropin-releasing factor (CRF) secretion by pituitary corticotrophic cells (Mezey et al., 1984).

Apart from the LH and PVN, medullary sympathetic premotor neurons contribute to glucose homeostasis by driving SPNs that control adrenaline release (Verberne and Sartor, 2010). Studies by Ritter and colleagues have identified the importance of catecholaminergic medullary neurons in mediation of the counterregulatory responses to glucoprivation (Ritter et al., 1998, 2001, 2006; Li et al., 2006, 2009). Systemic glucoprivation increases the firing rate of slow-conducting (<1 m/s) RVLM adrenal premotor medullospinal neurons (Verberne and Sartor, 2010), implying that they are C1 catecholaminergic cells (Schreihofer and Guyenet, 1997). Glucoprivation also elicits phosphorylation (Damanhuri et al., 2012), and expression of Fos (Ritter et al., 1998) and dopamine β-hydroxylase mRNA (Ritter et al., 2006) in RVLM C1 neurons. By contrast, neurotoxic ablation of C1 neurons eliminates the glucose response to the glucoprivic agent 2DG (Ritter et al., 2001; Madden et al., 2006). Interestingly, medullary orexinergic terminals (De Lecea et al., 1998; Peyron et al., 1998) make close appositions with RVLM C1 neurons (Puskas et al., 2010). Presumably, these close appositions arise from the orexin neurons labeled after injection of a neurotropic virus into the adrenal gland (Kerman et al., 2007). A subpopulation of these catecholaminergic neurons also expresses NPY (Li and Ritter, 2004). These neurons are located at the C1/A1 level and project rostrally to the hypothalamus (Verberne et al., 1999; Li and Ritter, 2004; Li et al., 2009) and are probably involved in the feeding response to neuroglucoprivation (Ritter et al., 2001; Li and Ritter, 2004). Finally, RVLM sympathetic premotor neurons make monosynaptic (McAllen et al., 1994; Zagon and Bacon, 1991; Oshima et al., 2008), glutamatergic (Morrison et al., 1989a; Morrison and Cao, 2000) connections with adrenal SPN (Morrison and Cao, 2000) to form a sympathoexcitatory pathway.

Studies using neuronal tracers have also identified direct projections from the PVN (Luiten et al., 1985) and the LH (Ter Horst et al., 1984; Allen and Cechetto, 1992) to parasympathetic motor neurons (Fox and Powley, 1986), particularly in the NTS/DMV area (Loewy et al., 1994). Furthermore, orexinergic terminals are found in the DMV (Date et al., 1999) and direct injection of orexin increases gastric motility (Krowicki et al., 2002) presumably mediated by an increase in parasympathetic nerve activity. An orexinergic input to the DMV has also been implicated in the increase in pancreatic parasympathetic nerve discharge produced by insulin-induced hypoglycemia (Wu et al., 2004). These findings suggest that the PVN and LH act as the major hypothalamic gateways for descending pathways that modulate glucose homeostasis (Luiten et al., 1985; Ter Horst and Luiten, 1987; Sim and Joseph, 1991).

The ARC/VMH and DMH neurons project to the DMV in the dorsal medulla, but they do not communicate with sympathetic premotor neurons in the ventral medulla. In fact, direct projections from the ARC/VMH and DMH to DMV motor neurons have been confirmed by anterograde (Ter Horst and Luiten, 1986; Sim and Joseph, 1991; Canteras et al., 1994) and retrograde (Ter Horst et al., 1984) tracer studies. However, there is no evidence for projections from ARC/VMH and DMH neurons to RVLM sympathetic premotor neurons. Although the studies by Borg and colleagues suggest that glucoprivation of VMH neurons induces glucagon, adrenaline and noradrenaline release, the microdialysis technique used in their studies is likely to have allowed diffusion of the glucoprivic agent throughout several hypothalamic regions, confounding the interpretation of these findings (Borg et al., 1995, 1997). Therefore, it is conceivable that additional inputs from ARC-VMH-DMH neurons to DMV neurons drive glucagon release, whereas adrenaline release is modulated in parallel by neurons in the LH (Yardley and Hilton, 1987) and PVN (Blair et al., 1996). Nevertheless, Chan and colleagues have clearly demonstrated that suppression of GABAergic drive in the VMH enhances the secretion of glucagon and adrenaline but not corticosterone in response to insulin-induced hypoglycemia (Chan et al., 2006). In STZ diabetic rats, blockade of VMH GABA receptors restores the glucagon response to hypoglycemia more effectively than the adrenaline response (Chan et al., 2011). Furthermore, these investigators have shown an inverse relationship between counterregulatory hormone release and VMH extracellular GABA (Zhu et al., 2010). On the other hand, Elmquist and colleagues have shown that reduction of VMH glutamatergic drive during hypoglycemia reduces the glucagon response to a greater extent than the adrenaline response (Tong et al., 2007).

### Intramedullary projections—evidence for independent glucose control in the brainstem

Several pieces of evidence suggest that the glucose counterregulatory network is confined to the brainstem, rather than involving the hypothalamus. Following decerebration (DiRocco and Grill, 1979) or obstruction of the cerebral aqueduct (Ritter et al., 1981), systemic glucoprivation with 2DG or injection of 5-thio-D-glucose (5TG) into the fourth ventricle elicits hyperglycemia, supposedly resulting from adrenaline release. However, these early studies assumed the involvement of adrenaline secretion based on glucose measurements alone, a role that could be fairly attributed to glucagon, as previously discussed in this review. Vagal afferent fibers conveying signals from the portal vein terminate onto the NTS and DMV neurons (Adachi et al., 1984; Berthoud et al., 1992).

Neurons in the DMV/NTS-A2 express Fos in response to hypoglycemia or glucoprivation (Ritter et al., 1998; Cai et al., 2001; Damanhuri et al., 2012) and a small proportion (21%) are glucose-sensing as based on electrophysiological characterization. This finding is supported by the presence of K_ATP_ channels and glucokinase in DMV (Balfour et al., 2006) and NTS (Briski et al., 2009) neurons. Nevertheless, activation of adrenal premotor neurons by an intrinsic drive from brainstem neurons cannot be disregarded. For instance, C1 sympathetic premotor neurons receive excitatory inputs from other brainstem nuclei including the NTS (Aicher et al., 1996), a structure which provides a high proportion of asymmetric synapses onto C1 neurons. By contrast, although sympathetic premotor neurons in the ventral medulla are activated by glucoprivation, evidence supporting the notion that they are intrinsically glucose-sensitive is poor.

Based on the evidence discussed here, it can be inferred that a rudimentary brainstem circuit is sufficient to counteract hypoglycemia and maintain life (DiRocco and Grill, 1979). It seems that NTS and DMV neurons constitute the first line of defense against hypoglycemia by mediating the release of glucagon. A proportion of these cells is intrinsically glucose sensitive, and receives input signals from vagal afferent neurons. However, whether the excitatory drive to adrenal premotor neurons following hypoglycemia or glucoprivation directly originates from brainstem neurons, or derives from descending hypothalamic projections is unknown. Moreover, if the former assumption is proven true, the question arises as to what is the role of the aforementioned hypothalamic circuitry. On the other hand, it can be speculated that the brainstem neurons that mediate the autonomic apparatus for glucose homeostasis, whereas hypothalamic neurons integrate complex behaviors such as feeding and arousal. This hierarchical structure of the neuroaxis adds a new dimension to the counterregulatory response to hypoglycemia. During the execution of these behaviors, it seems that the hypothalamic neurons can override the activity of brainstem neurons in order to adjust the autonomic outputs to a new metabolic demand. For instance, selective pharmacogenetic activation of ARC- AGRP neurons and optogenetic activation of orexinergic neurons elicit feeding (Krashes et al., 2011) and arousal (Adamantidis et al., 2007), respectively; behaviors that work in conjunction to increase glycemia.

## Efferent pathways for coupling to autonomic effectors

**Figure 3:**
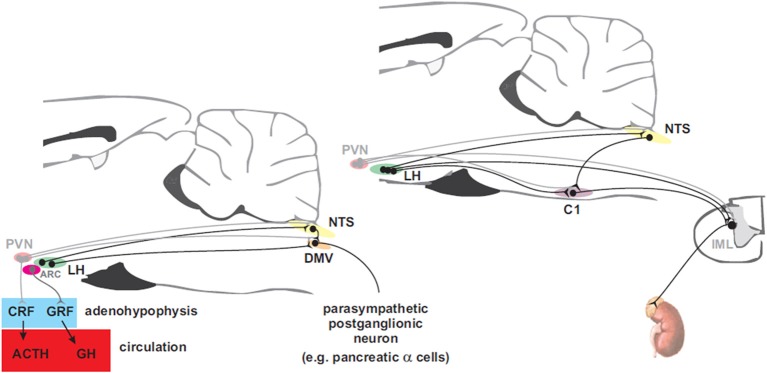
**Hypothalamic projections to the adenohypophysis and to parasympathetic motorneurons that innervate the pancreas (left panel) and to sympathetic premotor neurons that supply the adrenal gland (right panel)**. Neurons in the paraventricular nucleus of the hypothalamus (PVN) control the release of adrenocorticotropin (ACTH) into the circulation via corticotropin releasing factor (CRF) to promote secretion of cortisol from the adrenal cortex while arcuate (ARC) neurons control release of growth hormone (GH) via the release of growth hormone releasing factor (GRF). Preganglionic parasympathetic neurons of the dorsal motor nucleus of the vagus (DMV) receive inputs from neurons in the ARC and the lateral hypothalamus (LH). PVN and LH neurons send direct projections to the nucleus of the solitary tract (NTS) and to sympathetic preganglionic neurons in the intermediolateral cell column (IML). Adrenal catecholamine release is controlled by inputs from LH to C1 neurons in the rostral ventrolateral medulla (RVLM) that drive the adrenal sympathetic outflow.

The nervous system activates counterregulatory mechanisms to hypoglycemia in order to restore the blood glucose to normal levels. These mechanisms respond at different glycaemic levels (Cryer, 1997). In clinical studies the thresholds are: ~4.5 mM at which the pancreatic β-cell responds with a decrease in insulin secretion. At ~3.6–3.8 mM release of counterregulatory hormones (glucagon, adrenaline, GH, and cortisol) occurs. Furthermore, distinct subsets of neurons within the nervous system seem to selectively regulate these responses. In this section, we review the descending neural pathways and mechanisms controlling glucose counterregulatory hormones via sympathetic and parasympathetic motor neurons which originate in the hypothalamus and brainstem where the premotor neurons are found (Figure 3). Therefore, we first provide an insight of the neural mechanisms, at the motor level, that control insulin and glucagon secretion. Secondly, we discuss the neural control of adrenaline release, and finally, the modulation of GH and cortisol release.

### Neural control of pancreatic α- and β-cells

Insulin secreting β-cells and glucagon secreting α-cells are innervated by sympathetic and parasympathetic neurons (Gerich et al., 1976). The branches of the subdiaphragmatic vagus and of the splanchnic nerves form a mixed nerve connecting to the pancreas (Woods and Porte, 1974; Gerich et al., 1976). Electron microscopy has shown that both adrenergic and cholinergic terminals make contact with pancreatic islets (Madden and Sarras, 1989). These nerve terminals consist of both myelinated (Lever, 1964; Esterhuizen et al., 1968) and unmyelinated (Watari, 1968) fibers, which can be found in the periphery and within the center of the islets (Morgan and Lobl, 1968), in addition to ganglion cells (Honjin, 1956; Kobayashi and Fujita, 1969). In the nerve terminals found in the islets, electron microscopy reveals two major types of vesicles: cholinergic agranular vesicles and adrenergic granular cores (Richardson, 1964).

The pancreas receives parasympathetic cholinergic fibers in the vagus nerve, which originate in the DMV. *In vitro* and *in vivo* administration of acetylcholine induces insulin and glucagon secretion (Malaisse et al., 1967; Iversen, 1973a). These responses are mediated by activation of muscarinic receptors. Atropine reduces basal levels of insulin (Bloom et al., 1974a) and glucagon (Bloom et al., 1974b) induced by hypoglycemia and intravenous injection of arginine, respectively. *In vitro*, atropine also blocks the release of insulin and glucagon in response to acetylcholine (Malaisse et al., 1967; Iversen, 1973a). Stimulation of the vagus nerve elicits insulin and glucagon secretion in different species (Daniel and Henderson, 1967; Frohman et al., 1967; Kaneto et al., 1967, 1974), although bilateral section of the vagus fails to produce a sustained change in resting levels of insulin and glucagon (Hakanson et al., 1971; Bloom et al., 1974b). On the other hand, in humans, vagal section increases the periodicity of insulin secretion (Matthews et al., 1983). The cell bodies of vagal preganglionic neurons that regulate insulin and glucagon secretion are found in the DMV (Kalia, 1981). DMV neurons are located bilaterally ventral and medial to the nucleus of the solitary tract (NTS) in the dorsal surface of the caudal medulla, and are identified by choline acetyltransferase (ChAT) immunoreactivity (Takanaga et al., 2003; Llewellyn-Smith et al., 2013; Zheng et al., 2013). Injections of neurotropic pseudorabies virus into the pancreas retrogradely labels cell bodies of cholinergic neurons in the DMV (Jansen et al., 1997). Excitation of DMV neurons augments plasma insulin levels, whereas inhibition of these neurons reduces plasma insulin levels (Ionescu et al., 1983; Siaud et al., 1991).

Activation of sympathetic drive to the pancreas reduces insulin secretion and increases the secretion of glucagon. In experimental conditions, noradrenaline or adrenaline mimic the pancreatic response to sympathoexcitation, reducing insulin release and eliciting glucagon release (Coore and Randle, 1964; Karam et al., 1966; Porte and Williams, 1966; Iversen, 1973b). Similar results are observed following electrical stimulation of the splanchnic or the pancreatic mixed nerve, in the presence of atropine (Marliss et al., 1973; Bloom and Edwards, 1978). Anatomical findings further support this mechanism. The sympathetic projections to the pancreas originate in the celiac and superior mesenteric plexi, and converge at the greater and middle splanchnic nerves (Baron et al., 1985, 1988). Immunohistochemical studies show that the terminals of these nerves contain catecholamines (Miller, 1981). Thus, it is likely that these terminals belong to sympathetic postganglionic neurons, whose cell bodies are located in the sympathetic chain ganglia. The afferent inputs to these neurons are cholinergic (Feldberg, 1943; Feldberg et al., 1951; Oesch and Thoenen, 1973) and originate from SPN located in both the intermediolateral column (lamina VII) and the central autonomic area (lamina X) of the thoracolumbar spinal cord (Torigoe et al., 1985; Bacon and Smith, 1988; Pyner and Coote, 1994).

Apart from the noradrenergic and cholinergic inputs, neuropeptides also contribute to the innervation of the pancreatic islets. Neuropeptides released by the pancreatic nerve terminals probably also control islet function by modulating the release of insulin and glucagon (Ahren et al., 1986). Immunohistochemical studies have identified a variety of peptides in nerve terminals projecting to the pancreas. These include vasoactive intestinal polypeptide (VIP) (Bishop et al., 1980), cholecystokinin (CCK) (Rehfeld et al., 1980), gastrin releasing polypeptide (GRP) (Moghimzadeh et al., 1983), galanin (Dunning et al., 1986), NPY (Pettersson et al., 1987), calcitonin gene-related peptide (CGRP) (Pettersson et al., 1986), substance P and enkephalin (Larsson, 1979). In functional studies, VIP, CCK, and GRP appear to be excitatory while the inhibitory peptides are galanin, NPY, and CGRP and substance P and enkephalin produce diverse responses (Larsson, 1979).

### Adrenal sympathetic outflow

Adrenaline counters hypoglycemia by acting in the liver and pancreas. In the pancreas, adrenaline inhibits insulin release (Coore and Randle, 1964; Karam et al., 1966; Malaisse et al., 1967) and stimulates glucagon release (Iversen, 1973b; Gerich et al., 1974) whereas, in the liver, adrenaline activates gluconeogenesis and glycogenolysis (Exton, 1985; Pilkis et al., 1988; Kraus-Friedmann and Feng, 1996; Fabbri et al., 1998). In addition, adrenaline acts on skeletal muscle to reduce glucose uptake and also promotes lipolysis via an action at β_2_-adrenoceptors. Adrenaline is released by chromaffin cells in the adrenal medulla, under the control of SPN. Sympathetic projections to the adrenal gland are comprised of sympathetic pre- and post-ganglionic fibers (Carlsson et al., 1992). The preganglionic fibers to the adrenal gland are axons from a subset of SPNs, located in the T4–T13 segments of the spinal cord; the postganglionic fibers projecting to the adrenal gland originate from neurons in the sympathetic chain ganglion, and receive inputs from SPNs (Baron et al., 1988; Strack et al., 1988, 1989b). Neuroglucoprivation induces Fos expression in the adrenal medulla and in the intermediolateral cell column, primarily at spinal cord segments T7–T10, where adrenomedullary preganglionic neurons are found (Ritter et al., 1995). Neuroglucoprivation also activates the adrenal sympathetic outflow but not the renal sympathetic outflow (Niijima, 1975).

Systemic glucoprivation produces increases in levels of adrenaline and blood glucose, suggesting increase in the sympathoexcitatory drive to the adrenal gland (Ritter et al., 1995; Elman et al., 2004).

Adrenal-projecting SPNs can be functionally segregated according to electrophysiological properties and neurochemical phenotype. At least two subpopulations of SPN have been electrophysiologically differentiated: one group is involved in control of adrenaline secretion, while the other is related to the control of noradrenaline release (Morrison and Cao, 2000). The former subset of neurons is responsive to systemic glucoprivation, whereas the latter is exclusively inhibited by increases in blood pressure. The SPN clusters can be alternatively differentiated according to their neurochemical content. For instance, SPN that participate in cardiovascular regulation are immunoreactive for cocaine and amphetamine-regulated transcript peptide (CART); and in the adrenal, CART positive terminals selectively target noradrenergic chromaffin cells (Gonsalvez et al., 2010). On the other hand, enkephalin is likely to be a selective marker for adrenal-projecting SPN that control adrenaline release (Kumar et al., 2010). In fact, neuroglucoprivation produces c-Fos expression in prepro-enkephalin mRNA positive neurons, but fails to activate prepro-CART mRNA positive neurons (Parker et al., 2013). Although enkephalin is an inhibitory peptide used as a neurochemical marker, it does not imply that it inhibits chromaffin cells. Enkephalin produces variable responses in pancreatic islets (Green et al., 1983). Patients with type 1 or severe type 2 diabetes are at high risk of life-threatening hypoglycemia, due to a combination of intensive insulin treatment and impaired glucagon secretion (Halimi, 2010). Hypoglycemia usually occurs as a result of a mismatch between insulin dose, the amount of food consumed, and energy expended. Due to the destruction of pancreatic α-cells, in these patients adrenaline is the major glucose counterregulatory hormone secreted in response to hypoglycemia. Although slow-acting counterregulatory hormones contribute to rescue glucose levels (see below), the importance of adrenaline lies on the fact that it is the only remaining fast-acting counterregulatory hormone.

### Slow-acting glucose counterregulatory hormones

The slow-acting hormones, GH, and cortisol, contribute to glucose counterregulation by shifting metabolism of non-neural tissues away from glucose utilization (Schwartz et al., 1987). Cortisol activates fatty acid oxidation, gluconeogenesis and ketogenesis (Gerich et al., 1980). On the other hand, GH increases lipolysis, fatty acid oxidation and induces the insulin resistance noted in pregnancy (Barbour et al., 2002). Cortisol secretion is activated by adrenocorticotropic hormone (ACTH) release, which is modulated by CRF. Insulin-induced hypoglycemia is a stressor that produces large increases in plasma CRF (Engler et al., 1989) and ACTH (Pacak et al., 1995). CRF is synthetized by parvocellular neuroendocrine cells in the PVN of the hypothalamus and is released into the hypothalamo-hypophyseal portal system and transported to the anterior pituitary (adenohypophysis) where it stimulates corticotropes to secrete ACTH into the circulation. At the adrenal cortex, ACTH stimulates the synthesis of cortisol, glucocorticoids, mineralocorticoids, and dehydroepiandrosterone. Alternatively, hypoglycemia can also trigger the release of GH (Roth et al., 1963). This hormone is directly released by the adenohypophysis, and is primarily stimulated by the growth hormone-releasing factor (GRF) (Barinaga et al., 1985) produced in the ARC (Sawchenko et al., 1985). Nonetheless, growth hormone-secretagogues (Howard et al., 1996) and somatostatin (Plotsky and Vale, 1985), respectively, can stimulate and inhibit GH release.

## Conclusion

The preceding discussion provides a brief overview of the neural circuitry involved in the control of glucose homeostasis. However, in order to define how the neural pathways interplay to control glucose homeostasis, more detailed knowledge of the important neurons and their connections is still required. In contrast to brainstem neurons, we believe that further neuropharmacological and neurochemical characterization of hypothalamic neurons is necessary to understand their role in glucose control. Most of the current evidence relies on direct injections of drugs and neuronal tracers which, due to short projections within the hypothalamus, hamper the interpretation of the findings. Fortunately, new techniques are arising to overcome this issue. For instance, new pharmaco- and opto-genetic tools will help to determine the links between the neurochemical, pharmacological, and electrophysiological properties of hypothalamic neurons.

Two important questions about the role of hypothalamic neurons in the control of glucose homeostasis remain to be answered. The first is to define the hypothalamic gateway for downstream information, with an emphasis on the communication between the motor and premotor outputs and the glucose-sensing brainstem circuitry. Identification of neurons that control the release of adrenaline, glucagon, ACTH and GH will allow us to understand how these are controlled differentially and to determine the respective roles of the hypothalamic and brainstem nuclei. Secondly, a better knowledge of the hypothalamic circuitry involved in the control of blood glucose has yet to be elucidated. It will allow us to determine the structures and mechanisms underlying complex behaviors in response to hypoglycemia, which are related but distinct from autonomic and endocrine glucose homeostasis. For example, it can be speculated that brainstem neurons provide the essential output for autonomic responses while the hypothalamus integrates feeding, arousal and “fight or flight” behaviors associated with these responses. By answering these questions we may delineate the multi-layered neural apparatus that underpins glucose control and determine how it tunes different body systems to the brain's energy demand in mammals, a condition simply necessary for life.

### Conflict of interest statement

The authors declare that the research was conducted in the absence of any commercial or financial relationships that could be construed as a potential conflict of interest.
